# An Easily Used Phenomenological Magnetization Model and Its Empirical Expressions Based on Jiles–Atherton Parameters

**DOI:** 10.3390/ma15217592

**Published:** 2022-10-28

**Authors:** Guangming Xue, Hongbai Bai, Tuo Li, Chunhong Lu

**Affiliations:** 1 School of Mechanical Engineering and Automation, Fuzhou University, Fuzhou 350116, China; 2 Department of Unit Command, Officers College of PAP, Chengdu 610200, China; 3 Department of Automotive Engineering, Hebei College of Industry and Technology, Shijiazhuang 050091, China

**Keywords:** magnetization, first-order LTI system model, Jiles–Atherton model parameter, empirical equation, on/off type device

## Abstract

In this paper, a simple magnetization model convenient for engineering applications is presented based on the expressions of the first-order LTI system model. Considering the trade-off between the nonlinearity of anhysteretic magnetization and the hysteresis width, the proposed model employs two different equations with different magnetic field amplitudes. Furthermore, the proposed model utilizes the first-order LTI system model with a low magnetic field amplitude and a simple nonlinear function, based on the amplitude–frequency function, with a high magnetic field amplitude. Two important characteristic parameters for engineering applications, namely, amplitude and the equivalent phase lag, were exacted and analyzed to validate the computation precision of the proposed model. Then, the model was verified through comparisons to the validated Jiles–Atherton model. For easy use, similar to a physics-based model instead of a fitting method, empirical expressions for the model parameters were given, and applicable ranges of these equations were determined using the parameters of the Jiles–Atherton model. Finally, an example of the magnetization model applied to an on/off type device was computed to further verify the effectiveness of the proposed model with quite a simple expression.

## 1. Introduction

It seems that more complex magnetization models have become more popular in recent years [1,2,3,4,5,6,7,8,9,10,11,12], but this is not good news for the engineering applications of the models. These models directly used the classical model as a sub-model, or performed some adaptive improvements to the classical model to improve the applicability. With quite a strong nonlinearity, especially for the hysteresis, magnetization models are difficult to describe and solve. Moreover, it will bring disaster to the implementation of online control.

Current magnetization models are divided into two types: physics-based models and phenomenological models. Physics-based models consider the magnetizing mechanism, and most parameters of this type of model have certain physical means. A commonly used physics-based magnetization model was proposed by Jiles and Atherton [13,14,15,16], named the Jiles–Atherton (J–A) model. The quasi-static J–A model utilizes five equations, including the differential element and Langevin function, to describe magnetization when complicated expressions provide no possibilities for any analytical solution. Even a high-precision numerical solution was not easily achieved in previous papers when the magnetic field intensity was not high enough [17,18,19]. The Smith free-energy model [20,21] employs the mean value of the local magnetization derived from the quadratic Helmholtz model and executes a double integral on the core function. Although it has a simpler expression than the J–A model, the Smith free-energy model is hardly solved, and its accuracy relies on the discontinuous core function [22,23]. Without analyzing the magnetizing process, a phenomenological model generally has more concise expressions than a physics-based model. The Preisach model [24], the Duhem model [25,26] and the neural network model [6,27,28,29,30,31] are the three most commonly used phenomenological models. The Preisach model utilizes a surface integral to describe magnetization, while the Duhem model uses a first-order differential equation and is more easily imposed with identification algorithms and compensation control. The neural network provides many choices for describing magnetization while considering the trade-off between high precision and high efficiency. Besides the above models, physics-based models, such as the Armstrong model [32,33] and the Bouc–Wen model [34,35], and phenomenological models, such as the polynomial model [36], the Krasnoselskii–Pokrovskii (KP) model [37] and the Prandtl–Ishlinskii (PI) model [38,39], have also been employed to describe magnetization [40].

The abovementioned magnetization models have been commonly used in many areas; however, two key problems in engineering applications have not been solved. Firstly, these models have not reached the simplification degree required by engineering applications. Among the abovementioned models, the Duhem model, the Bouc–Wen model (a nonlinear second-order differential equation) and the polynomial model have the most concise forms, while the sign function or absolute value function always remains to distinguish the rising and falling processes that the first derivative at the turning point indicate are not continuous. It seems that these models are “simplified while not completely simplified” and that they are unhelpful for future control. This problem is difficult to solve, resulting in many studies directly covering up the magnetization–magnetic field sub-models and constructing a more macroscopic output–input relationship. Besides the engineering expectation of simplification described below, the verification index for the magnetization model is highly confusing. It seems that either the model supporter or the user does not know how precise the model is required to be or what characteristic parameter represents “precision”. In many studies, the models have only been verified by putting together the model and the test results, which indicated that the model is in good agreement with the experiment, as the results looked similar from the perspective of the reader. The unclear requirements of magnetization models have led to the blindness of model applications.

**Figure 1:**
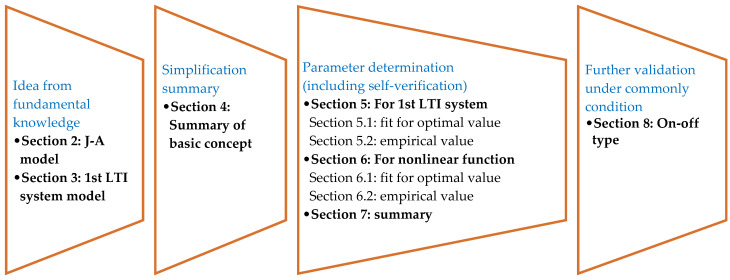
Structure of this article.

This paper proposed a new model for static magnetization, taking inspiration from the first-order LTI system model. The proposed model fully utilizes the different main features of magnetization under various magnetic field amplitudes on the premise of meeting the strict requirements requested by engineering applications for the computation of amplitude and equivalent phase lag. The model is easily controlled, as it employs the first-order LTI system model under a low magnetic field and a nonlinear function based on the amplitude–frequency function of the first-order LTI system under a high magnetic field. The research idea and framework of the article are shown in Figure 1. In Section 2 and Section 3, some necessary information on the J–A model and 1st LTI system model is given and analyzed. Then, the simplification idea and format are supplied in Section 4. The parameters of the simplified model were determined in two methods, respectively, from extra knowledge of the maximum magnetization and just from J–A parameters. Additionally, optimal values and empirical equations for parameter determination are given in Section 5, Section 6 and Section 7 for best computational effects or convenience. At last, a commonly faced condition of an on-off type device is discussed in Section 8 to give a necessary supplement. Comparisons of the calculated results between the proposed model and verified J–A model were carried out to validate the computation precision of the proposed model. From comparisons, the model reached acceptable computation precisions on the amplitude and phase lag (equivalent) of the magnetization with quite simple expression.

## 2. Jiles–Atherton Hysteresis Model

### 2.1. Description and Solution

Xue et al. summarized the various expressions of the Jiles–Atherton hysteresis model cited in different references, and they proposed a reasonable expression based on the mathematical properties of the Langevin function and the actual magnetization process [15,35]. The sorted formula is expressed as

(1) $$\left\{\begin{array}{l} M_{an}=M_{s}[\coth(\frac{H+\alpha M_{an}}{a})-\frac{a}{H+\alpha M_{an}}]\\ \frac{dM}{dH}=\frac{\delta kc\frac{dM_{an}}{dH}+\delta_{M}(1-c)(M_{an}-M)}{\delta k-\alpha \delta_{M}(1-c)(M_{an}-M)} \end{array}\right.$$

where *M_s_* is the saturation magnetization; *M_an_* is the anhysteretic magnetization; *α* represents the quantified domain interactions; *a* is the shape parameter for *M_an_*; *c* is the reversibility coefficient; *k* is the average energy required to break pinning sites; and *δ* is the sign function of the derivative of the magnetic field strength with respect to time, expressed by sign(d*H*/d*t*) and equal to 1 when d*H*/d*t* > 0 and equal to −1 when d*H*/d*t* < 0. *δ_M_* is employed to guarantee positive incremental susceptibilities; it is equal to 0 under the condition of sign[(d*H*/d*t*)(*M_an_* − *M*)] < 0, and it is equal to 1 under any other condition.

The analytical solution of Equation (1) cannot be obtained, and a high-precision solution is not easy. Ref. [15] supplied a fast, high-precision solving method, where *M_an_* is solved by the fixed-point iteration, and then *M* is solved using fourth-order Runge–Kutta equations. The solving method is expressed by

(2) $$\left\{\begin{array}{cc} M_{an}^{\left(i\right)}= & M_{s}\left[\coth(\frac{H+\alpha M_{an}^{\left(i-1\right)}}{a})-\frac{a}{H+\alpha M_{an}^{\left(i-1\right)}}\right]\\ M_{n+1}= & M_{n}+h(k_{1}+2k_{2}+2k_{3}+k_{4})/6\\ k_{1}= & \psi (H_{n},M_{n})\\ k_{2}= & \psi (H_{n}+h/2,M_{n}+hk_{1}/2)\\ k_{3}= & \psi (H_{n}+h/2,M_{n}+hk_{2}/2)\\ k_{4}= & \psi (H_{n}+h,M_{n}+hk_{3}) \end{array}\right.$$

where *M_an_*^(*i*)^ is the value of *M_an_* after *i* times of iterations. The iterative initial value can be imposed as *M_an_*^(0)^ = 50 *H*, and the number of iterations *i*_max_ ≥ 3, or *M_an_*(0) is set as any constant, and *i*_max_ ≥ 5. In Equation (2), the differential function value *ψ*(*H*,*M*) = $\frac{dM}{dH}=\frac{\delta kc\frac{dM_{\mathrm{an}}}{dH}+\delta_{M}(1-c)(M_{\mathrm{an}}-M)}{\delta k-\alpha \delta_{M}(1-c)(M_{\mathrm{an}}-M)}$.

**Figure 2:**
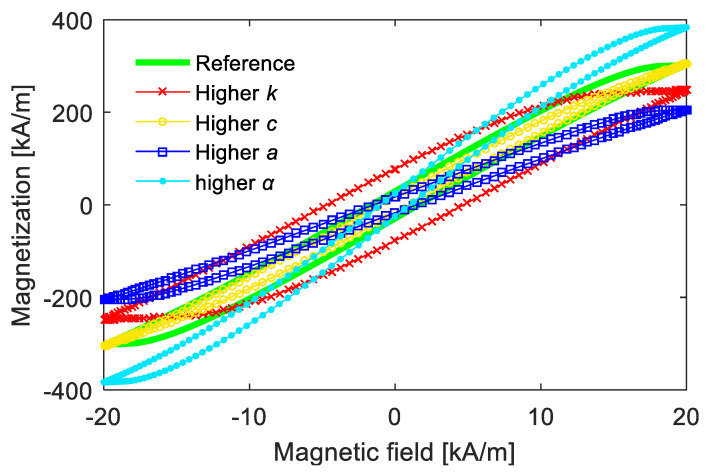
Examples of magnetic hysteresis computed from Jiles–Atherton model.

Figure 2 shows the description effect of the Jiles–Atherton model on magnetic hysteresis with different parameters. Roughly speaking, the values of *k* and *c* mainly influence the width of the hysteresis loop, and an increased *k* value or a decreased *c* value is helpful for a narrower hysteresis curve. The values of *a* and *α* mainly affect the slope of anhysteretic magnetization, and one obtains steeper slopes with higher values of *α* or lower values of *a*. As the scale parameter of *M_an_*, the saturation magnetization *M_s_* mainly influences the magnification degree of the magnetization value.

### 2.2. Key Characteristic Parameters in Time-Domain Performance

The nonlinear variation and the width of the hysteresis loop are the key characteristics of magnetization in engineering applications, while the performance of these two characteristics has a trade-off relationship under fixed parameters. That is, a strong nonlinear variation in anhysteretic magnetization is always combined with a relatively narrow hysteresis curve when the magnetization amplitude is high. However, when the magnetization amplitude is low, anhysteretic magnetization shows a weak nonlinear variation, while the hysteresis loop is quite wide.

**Figure 3:**
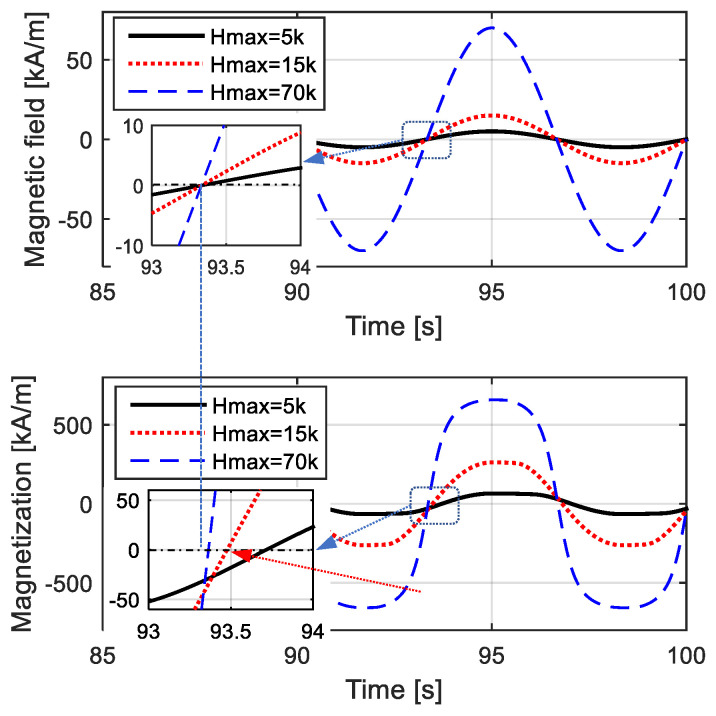
Time-domain curves of *H* and *M* with different amplitudes and different lagging phases.

**Figure 4:**
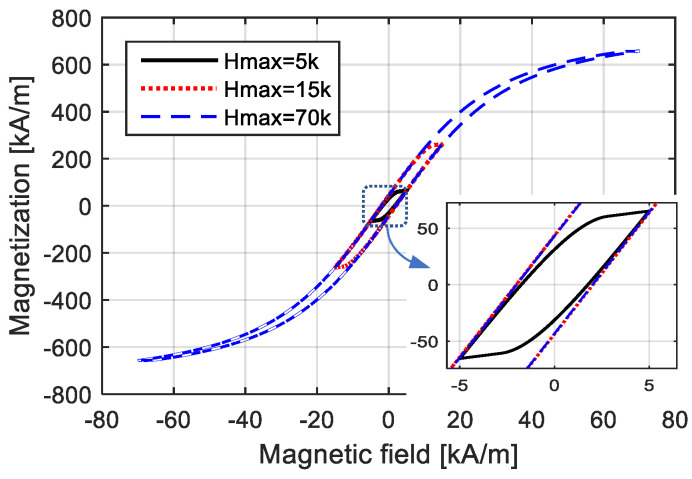
*M*–*H* curves with different amplitudes.

The amplitude and width of the *M*–*H* loop approximately correspond to the maximum value and the equivalent phase lag of time-domain magnetization. To demonstrate the trade-off relationship between the nonlinear variation and the width of the hysteresis loop, time-domain curves of the magnetic field and magnetization are shown in Figure 3, and the corresponding *M*–*H* curves are shown in Figure 4. The “phase lag” of the magnetization was not directly defined in the hysteresis while could be reflected by the width of hysteresis loop, coercivity and remanence. Just like the harmonic response, equivalent phase lag in the *M*–*H* model can be determined by the coordinate difference of *M* and *H* intersections with horizontal axis, as shown in Figure 3. For the *M*–*H* curve, the equivalent phase lag can be reflected by the proportion of the *H* value at *M* = 0 to *H*_max_.

When employing a low-amplitude magnetic field, shown by the solid black lines in the figures, the loop was wide, indicating that the time-domain curve of magnetization *M* had an obvious equivalent phase lag compared with the time-domain curve of the magnetic field *H*. Meanwhile, the variation in anhysteretic magnetization was approximately linear, indicating that the time-domain curve of magnetization *M* was quite similar to a standard sinusoidal curve, and the *M*–*H* curve was not obviously curved. On the contrary, when the *H* value was quite high, as shown by the blue dotted lines in Figure 3 and Figure 4, the loop of the *M*–*H* curve seemed narrow, indicating that *M* had a few equivalent phase lags compared to *H* in the time-domain; the magnetization variation was quite nonlinear, indicating that the time-domain curve of *M* was completely different from a sinusoidal curve; and the *M*–*H* curve was obviously curved.

Generally speaking, when the magnetic field strength is high, the variation in the nonlinearity of anhysteretic magnetization is strong, while the equivalent phase lag of magnetization to the magnetic field is little. When the magnetic field strength is low, the equivalent phase lag is high, while the variation in anhysteretic magnetization seems to be linear. This provides the new idea that only the main characteristic of hysteresis is considered to simplify the hysteresis model in engineering applications. Moreover, the trade-off between the nonlinear variation in *M_an_* and the width of the hysteresis loop provides the possibility for this type of simplification.

## 3. Response of First-Order Linear Time-Invariant (LTI) System

### 3.1. Description and Solution

The first-order LTI system is the simplest LTI system. Inheriting the parameters of an *RL* series circuit model, the first-order LTI system, describing the relationship of magnetization *M*(*t*) and the magnetic field *H*(*t*), can be written as

(3) $$L\frac{dM(t)}{dt}+RM(t)=M_{s}H(t)$$

where *L* and *R* are two parameters independent of time *t*. The maximum value of *M*(*t*) is constrained by *R*, *L*, and saturation magnetization *M_s_*.

According to the theory of the first-order LTI system, the steady-state response under *H*(*t*) = *H*_max_sin(*ωt*) is

(4) $$M(t)=\frac{M_{s}}{\sqrt{{\left(L\omega \right)}^{2}+R^{2}}}H_{\max}\sin(\omega t-\varphi )$$

where *φ* is the equivalent phase lag of *M*(*t*) compared to *H*(*t*). *φ* satisfies the conditions that tan*φ* = *ωL*/*R* and 0 ≤ *φ* < π.

From the solution expressed by Equation (4), $M_{s}H_{\max}/\sqrt{{\left(L\omega \right)}^{2}+R^{2}}$ determines the amplitude, and φ determines the lagging phase of steady-state magnetization.

From Appendix A, the first-order LTI system is capable of describing the hysteresis characteristics of the independent and dependent variables, and it can predict various hysteresis characteristics by adjusting *R* and *L*.

### 3.2. Description of Magnetic Hysteresis by the First-Order LTI System Model

According to the results shown in the above sections, it seems feasible to predict the magnetic hysteresis using the first-order LTI system model instead of the Jiles–Atherton model. Furthermore, various hysteresis loops with different widths or amplitudes can also be achieved by adjusting the values of R or L, similar to the influences of the parameters of the Jiles–Atherton model on the hysteresis.

**Figure 5:**
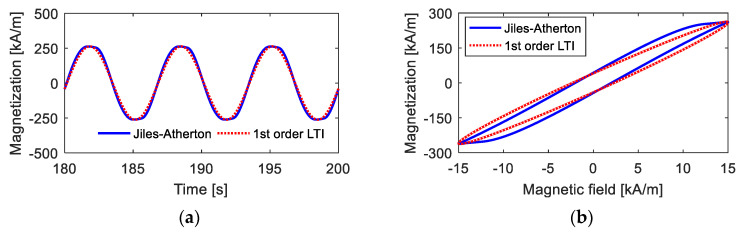
Magnetization curves under low magnetic field intensity calculated from the Jiles–Atherton model and the 1st-order LTI system model: (**a**) time-domain curves; (**b**) *M*–*H* curves.

Figure 5 shows the description effects of the first-order LTI system model, including the time-domain and *M*–*H* curves, on the hysteresis loop when the amplitude of the magnetic field intensity is low. From the calculation results, it is observed that the first-order LTI system can effectively predict the amplitude and width of the hysteresis loop as long as the appropriate parameters are provided. For the corresponding time-domain curve of magnetization, the curve computed by the Jiles–Atherton model is quite similar to standard sine curves, and the first-LTI system model with the appropriate parameters provides a good predicting effect. The main deviations occur during the variation process from 0 to maximum (or minimum) magnetization. These deviations are not so apparent and are further reduced when calculating the final independent response with other linear or nonlinear links being superimposed.

**Figure 6:**
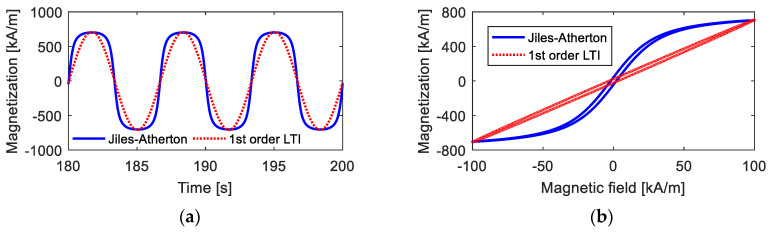
Magnetization curves under high magnetic field intensity calculated from the Jiles–Atherton model and the 1st-order LTI system model: (**a**) time-domain curves; (**b**) *M*–*H* curves.

However, when the maximum value of the magnetic field intensity Hmax is high, as shown in Figure 6, the deviations between the results calculated from the first-order LTI system model and those calculated from the Jiles–Atherton model are quite high. With a high magnetic field, the time-domain curve of magnetization deviates far from standard sinusoidal curves. Moreover, under this condition, anhysteretic magnetization *M_an_* has a strong nonlinearity, indicating that the hysteresis loop has a large bending, and the first-order LTI system cannot describe a curved hysteresis loop, as analyzed in Section 2.2.

### 3.3. Introduction of Amplitude–Frequency Function

Although the first-LTI system model fails to describe the nonlinear characteristic of *M_an_*, mainly the saturation nonlinearity, the amplitude–frequency characteristic of the specific first-order LTI system model shows saturation characteristics. Therefore, the amplitude–frequency function can be employed to predict the nonlinear relationship between *M* and *H* when the amplitude of the magnetic field is high.

Consider the function of $K_{x}x/\sqrt{x^{2}+f^{2}(x)}$, one obtains $\underset{x\to \infty }{\lim}K_{x}x/\sqrt{x^{2}+f^{2}(x)}$ $=\underset{x\to \infty }{\lim}K_{x}/\sqrt{1+{\left[f(x)/x\right]}^{2}}=K_{x}$ under the condition that $\underset{x\to \infty }{\lim}f(x)/x=0$. Take this function to describe magnetization as

(5) $$M(t)=\frac{M_{s}}{\sqrt{H{\left(t\right)}^{2}+f^{2}\left[H(t)\right]}}H(t)$$

where *f*[*H*(*t*)] is an undetermined function meeting $\underset{x\to \infty }{\lim}f(x)/x=0$.

In essence, Equation (5) is a simplified fitting function ignoring the hysteresis loop to approximate *M*, which is similar to using *M_an_* to approximate *M*. The difference here is that *M_an_* is expressed implicitly by the Langevin function, while Equation (5) supplies quite a simple explicit equation.

## 4. Simple Hysteresis Model

A simplification idea can be realized from the discussions in Section 3.1, Section 3.2 and Section 3.3. When the magnetic field intensity is low, the hysteresis loop of magnetization is wide, the variation in the nonlinearity of the anhysteretic magnetization is weak, and magnetization can be illustrated by the first-order LTI system model expressed as Equation (3). In contrast, when the magnetic field intensity is high, the variation in the nonlinearity is strong, the loop is narrow, and magnetization can be described using the simple nonlinear function shown in Equation (5). Then, one obtains the magnetization model expressed by

(6) $$\left\{\begin{array}{ll} L\frac{dM(t)}{dt}+RM(t)=M_{s}H(t), & if\;H_{\max}\leq H_{r}\\ M(t)=\frac{M_{s}\cdot H(t)}{\sqrt{H{\left(t\right)}^{2}+K_{an}^{2}}}, & if\;H_{\max}>H_{r} \end{array}\right.$$

where *K_an_* is a parameter only related to the material properties (the simplest form of Equation (5)), and *H_r_* is the split magnetic field used to distinguish the application scopes of the two sub-models. The value of r in *H_r_* is a real number belonging to [0, 1], and *H_r_* represents the required magnetic field intensity, achieving magnetization with a value of *r·M_s_*. From the definition of *H_r_*, one obtains *H*_0_ = 0, *H*_1_ = +∞.

The hysteresis model expressed by Equation (6) considers the width while neglecting the bending state of the hysteresis loop under a low magnetic field intensity. From the point of view of the time-domain magnetization curve, the model considers the phase lag of magnetization while neglecting the variation process from 0 to the maximum or minimum value. On the contrary, when the magnetic field intensity is high, the model considers the bending state while neglecting the width of the loop, which means that the model considers the variation process while neglecting the equivalent phase lag. The proposed model was verified from the comparisons of the calculated results with any verified magnetizations from verified models or tests. Considering the verified model is more convenient than tests to acquire magnetization results of various materials, the physics-based Jiles–Atherton model with abundant parameters was chosen to generate accurate magnetization values for comparisons.

## 5. First-Order LTI System Sub-Model under Low Magnetic Field Intensity

### 5.1. Optimal Parameter for Amplitude

The determination of the values of *R* and *L* is a complex parameter optimization problem. It was expected to achieve the objective of the amplitude and equivalent phase lag of the computed magnetization being close to those calculated using the Jiles–Atherton model. Furthermore, considering the importance of amplitude in engineering applications, the calculation accuracy of the amplitude should be guaranteed first when there is a trade-off between the calculation accuracies of the amplitude and equivalent phase lag.

Moreover, the equivalent phase lag could be determined by using Equation (4) to directly fit the calculated results of the Jiles–Atherton model. Of course, the direct fitting method, using the least square fitting method, takes into account both the amplitude and phase lag, and it indicated that the amplitude of magnetization was not determined so accurately.

To acquire a more accurate amplitude, the values of maximum magnetization *M*_max_, the maximum magnetic field intensity *H*_max_, and the equivalent phase lag *φ* were first extracted. Based on the amplitude–frequency and phase–frequency relationships shown in Equation (4), the optimal parameter value can be directly computed from $M_{\max}=\frac{M_{s}}{\sqrt{{\left(L\omega \right)}^{2}+R^{2}}}H_{\max}$ and tan*φ* = ω*L*/*R*, where one obtains

(7) $$\left\{\begin{array}{l} \hat{L}\cdot \omega =\frac{M_{S}H_{\max}}{M_{\max}}\sin\varphi \\ \hat{R}=\frac{M_{S}H_{\max}}{M_{\max}}\cos\varphi \end{array}\right.$$

In short, from the three steps of (1) direct fitting to extract the equivalent phase lag, (2) extracting the maximum value, and (3) computing, the optimal values of *R* and *L* for the first-order LTI system model were determined.

**Figure 7:**
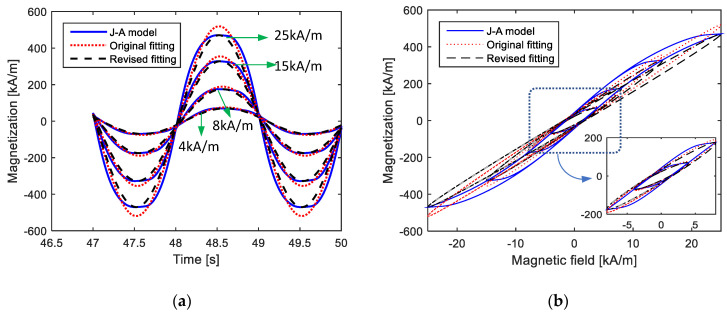
Comparisons of calculated magnetization curves from Jiles–Atherton model, the 1st LTI system model with parameters from directly fitting and the 1st LTI system model with parameters from fitting and extracting (under fixed parameters of *k* = 2 kA/m, *a* = 12 kA/m, *c* = 0.2, *α* = −0.001 and *M_s_* = 80 kA/m during different amplitudes of magnetic field intensity): (**a**) time-domain curves; (**b**) *M*–*H* curves.

Figure 7 shows the computation effects of the different parameters from direct fitting and Equation (7), labeled as “Revised fitting”. From the results, it can be observed that the first-order LTI system model with the appropriate parameters effectively described the amplitude and equivalent phase lag of magnetization under a low *H*_max_. Furthermore, compared to direct fitting, revised fitting with the parameters computed from Equation (7) preserved the amplitude and equivalent phase lag more precisely. This could indicate that Equation (7) achieved the perfect amplitude and equivalent phase lag by sacrificing the calculation accuracy in the changing process of magnetization from 0 to maximum or minimum, which is not so important in engineering applications.

### 5.2. Empirical Equations for the Parameters

Equation (7) can predict the exact values of the amplitude and the equivalent phase lag, and the equation relies on a fitting method to predetermine the equivalent phase lag. Here, some empirical expressions are provided for easy use.

#### 5.2.1. Function Format

**Figure 8:**
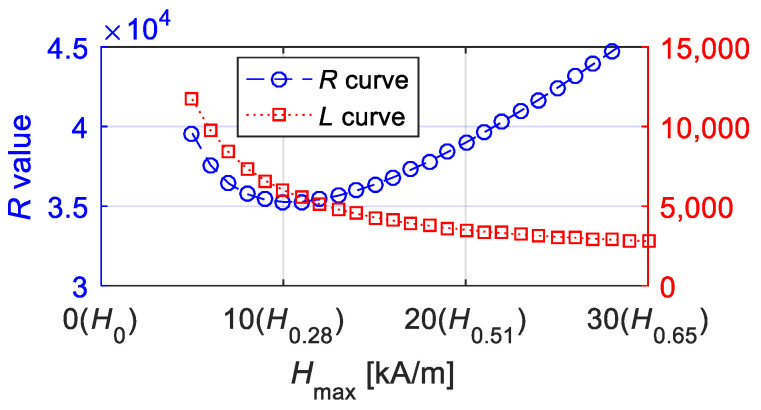
Optimal values of *R* and *L* under fixed parameters of *k* = 2 kA/m, *a* = 12 kA/m, *c* = 0.2, *α* = −0.001 and *M_s_* = 80 kA/m during different amplitudes of magnetic field intensity (the Abscissa axis gave the values of amplitude and *r* value of *H_r_* simultaneously).

The values of *R* and *L* computed from the equivalent phase lag and *M*_max_ are time-independent and related to the material properties. The main concern is that they are not independent of the maximum magnetic field intensity *M*_max_. Figure 8 provides the optimal values of *R* and *L* at different values of *H*_max_.

To express the variables of *R* and *L* in simple and accurate formulae, the following can be observed: (1) *R* or *L* is better expressed by a function of *H*_max_ while not being an *H*_max_-independent variable. That is, it is better to predetermine the value of *H*_max_. (2) To meet $\underset{H_{\max}\to \infty }{\lim}M_{s}H_{\max}/\sqrt{{\left(L\omega \right)}^{2}+R^{2}}=M_{s}$, the constructed functions of *L* and *R* can follow (*Lω*)^2^ + *R*^2^ = *f*^2^(*H*_max_) + *H*_max_^2^ for a similar amplitude–frequency function indicating that $\underset{x\to \infty }{\lim}K_{x}x/\sqrt{x^{2}+f^{2}(x)}$ = *K_x_*. (3) The equivalent phase lag *φ* decreases with an increase in *H*_max_ until 0, and then, one obtains tan*φ* = *ωL*/*R*. Based on (2), the expression of *R* must include the linear term of *H*_max_ and meet $\underset{H_{\max}\to \infty }{\lim}R/H_{\max}=1$. Moreover, the expression of *L* should meet $\underset{H_{\max}\to \infty }{\lim}L\omega /H_{\max}=0$. (4) The power function and constant are used, while the other function forms are not introduced as far as possible for simple expressions.

Based on the curve shape and the above principles, the functions of the variables *R* and *L* were determined as follows:

(8) $$\left\{\begin{array}{l} \hat{L}=A_{L}H_{\max}^{B_{L}}/\omega_{L}\\ \hat{R}=H_{\max}+C_{R}H_{\max}^{E_{R}}+D_{R} \end{array}\right.$$

where *A_L_* and *B_L_* are the parameters of *L*, and *C_R_*, *D_R_*, and *E_R_* are the parameters of *R*. All parameters are independent of time, and they are only related to the material properties, such as *M_S_* and *a*. To meet ${\left.\frac{M_{s}H_{\max}}{\sqrt{{\left(L\omega \right)}^{2}+R^{2}}}\right|}_{H_{\max}=0}=0$ and $\underset{H_{\max}\to \infty }{\lim}\frac{M_{s}H_{\max}}{\sqrt{{\left(L\omega \right)}^{2}+R^{2}}}=M_{s}$, the power term satisfies *B_L_* ∈ [0, 1) and *E_R_* ∈ [0, 1).

*ω_L_* was introduced to eliminate the influence of the waveform and the frequency of the magnetic field intensity *H*. In essence, *ω_L_* was used to remove the influence of d*H*/d*t* on the calculated magnetization. The calculation results of the static Jiles–Atherton model are only related to the amplitude of *H* and not to the increase in *H*, while the first-order LTI system model must be independent of the increase in *H*. To remove the influence of the frequency or waveform, the expression of 1/*ω_L_* can be added to *L*, and then *L*·*ω* can be formed as a parameter independent of the increase in *H*. The value of *ω_L_* can be easily determined from several empirical fittings. For a sinusoidal magnetic field, *ω_L_* = *ω*. For aperiodic signals or rising and falling signals commonly used in switching devices, 1/*ω_L_* can be determined without considering the frequency and only considering the influence of the waveform. So, one advantage of employing the first-order LTI system model to describe the magnetic hysteresis is that the amplitude and the phase of the linear steady system response can naturally reflect the influence of the frequency loss and then easily describe the magnetic loss at a high frequency. Instead, the effect of the frequency should be eliminated in the case of static hysteresis, or the hysteresis caused by the frequency should be ignored.

#### 5.2.2. Parameter Determination

The five parameters *A_L_*, *B_L_*, *C_L_*, *D_R_*, and *E_R_* were only determined by the properties of the material, referring to *α*, *a*, *c*, and *k* in the Jiles–Atherton model (*M_S_* was previously considered). For a specific material, the five parameters are invariant parameters and can be easily determined by any fitting method.

Here, an easy-to-use empirical equation suitable for various materials was presented. It is difficult to determine these multivariate functions, especially when the function form is unknown and there is no clear fitting direction. The univariate fitting of a single independent variable was executed to determine several univariate functions, and then the multivariate function was constructed by the linear combination of these univariate functions.

(1) Select one parameter as the independent variable and fix the other variables as any value. Calculate the magnetization values using the Jiles–Atherton model or any other verified hysteresis model based on the different values of the specified independent variable.
(2) Extract the maximum value of magnetization *M*_max_ and determine the equivalent phase lag *φ* via direct fitting.
(3) Calculate the optimal values of *R* and *L* under variable parameters using Equation (7) based on the obtained *M*_max_ and φ under different values of *H*_max_.
(4) Determine the optimal values of *A_L_*, *B_L_*, *C_L_*, *D_R_*, and *E_R_* using the fitting method based on Equation (8).
(5) Fit the functional relationship between the optimal values of *A_L_*, *B_L_*, *C_L_*, *D_R_*, and *E_R_* and the specified independent variable. The computational precision of amplitude is guaranteed with priority.
(6) Change the selected variable, return to (1), and then repeat (1)–(6) to determine the functional relationship (univariate) between the optimal values of *A_L_*, *B_L_*, *C_L_*, *D_R_*, *E_R_*, and the other parameters.
(7) Employ the linear combination method to transform multiple univariate functions into a multivariate function.

An example is given in Appendix B. Other parameters are determined successively according to the above method. Finally, one obtains

(9) A^L=−16k2+6.74×104k+8364a+2.55×108e−7.2c−1.17×1011α2−2.68×109α−1.8×108B^L=1.43×10−8k2+1850/a+0.21e2.7c+312α2+2α−1.5C^R=4.86k2+0.2a2−4500a+8.65×107c3−9.56×107c2+2.13×107c−1.8×108α+9.23×106D^R=−0.0062k2+34.5k+2.3×10−4a2−4.33a−3.23×107c10.6+5×106α+21.3×105α−2.23×104E^R=1.28×10−7k2−8.34×10−4k−5.1×10−9a2+1.26×10−4a+2.6c3.4−0.17

By substituting Equations (8) and (9) into Equation (3), one obtains the first-order LTI system sub-model, which is effective under the magnetic field intensity with a low amplitude.

#### 5.2.3. Verification under Fixed Values

**Figure 9:**
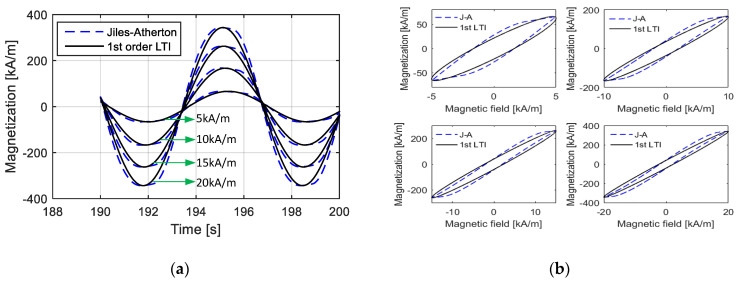
Comparisons of calculated magnetization curves from the Jiles–Atherton model and the 1st LTI system model under fixed parameters (*k* = 2 kA/m, *a* = 12 kA/m, *c* = 0.2, *α* = −0.001 and *M_S_* = 80 kA/m) during various magnetic field intensities with low amplitudes: (**a**) time-domain curves; (**b**) *M*–*H* curves.

Figure 9 shows the calculation effect of adopting the first-order LTI system model using empirical equations under the parameters of *k* = 2 kA/m, *a* = 12 kA/m, *c* = 0.2, *α* = −0.001, and *M_S_* = 80 kA/m. The maximum values of the magnetic field intensity were taken as *H*_0.083_ = 5 kA/m, *H*_0.209_ = 10 kA/m, *H*_0.327_ = 15 kA/m, and *H*_0.429_ = 20 kA/m, where the corresponding maximum magnetization values at these magnetic field intensities reached 0.083 *M_S_*, 0.209 *M_S_*, 0.327 *M_S_*, and 0.429 *M_S_*, respectively. The parameters were calculated using Equation (9). Compared to the Jiles–Atherton model, the first-order LTI system model could effectively predict the amplitude and equivalent phase lag of magnetization, which is consistent with prior designs. The prediction effect deteriorated with an increase in *H*_max_. Moreover, the deviation was mainly embodied in the change process between 0 and maximum or minimum and 0.

**Figure 10:**
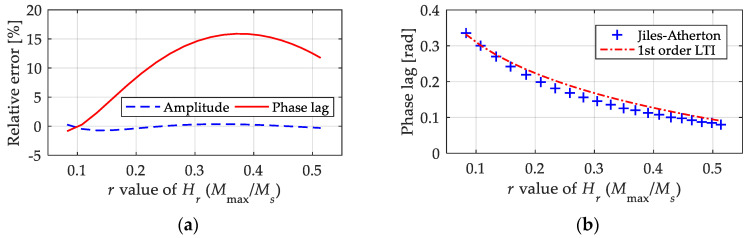
Relative errors of the 1st LTI system model in calculating the amplitude and equivalent phase lag under fixed parameters (*k* = 2 kA/m, *a* = 12 kA/m, *c* = 0.2, *α* = −0.001 and *M_S_* = 80 kA/m): (**a**) relative errors; (**b**) absolute value of equivalent phase lag.

We extracted the relative errors of the first-order LTI system model when computing the amplitude and equivalent phase lag, and they are shown in Figure 10, in which the abscissa variable is used as the *r* value of *H_r_* to represent the magnetic field intensity with an amplitude from 5 kA/m to 25 kA/m. From the results of the relative errors, it was observed that the first-order LTI system model could describe the amplitude of magnetization with high precision, as the relative error was less than 2%. In contrast, the proposed first-order LTI system model failed to precisely predict the equivalent phase lag, as the relative error exceeded 15% in some cases. Combined with the absolute value of the equivalent phase lag shown in Figure 10 and the time domain curves shown in Figure 9, this level of calculation accuracy of the equivalent phase lag is acceptable in engineering applications. In fact, the calculation deviation will be weakened when more links are added to the final output.

Area of hysteretic loop is an important parameter to indicate the energy loss per period. As the amplitude and equivalent phase lag can be acquired with low deviations by the proposed model, it can be concluded approximately that the loop area can be calculated effectively. Table 1 gives the calculated areas of hysteretic loop from the two models. From the calculated results, the first-order LTI system model had relative error not higher than 4.0% compared to the proposed model in calculating the loop area accurately.

#### 5.2.4. Parameter Applicability

**Figure 11:**
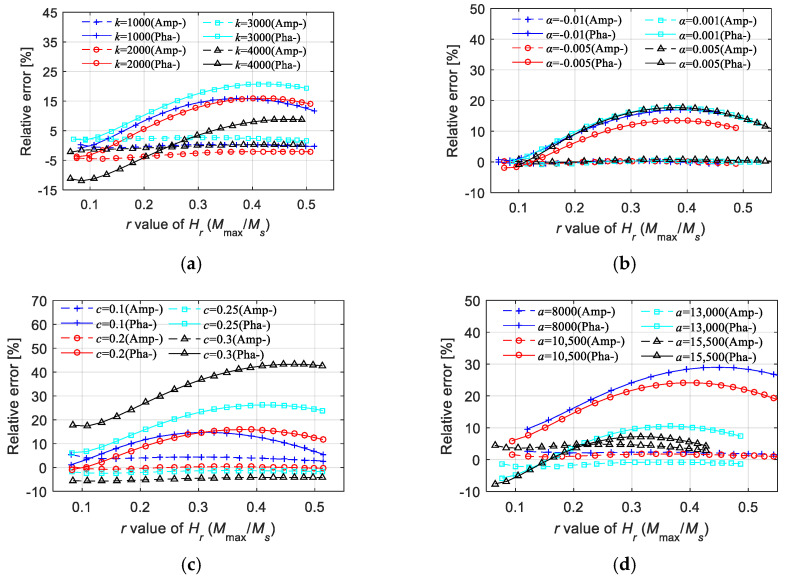
Relative errors of the 1st LTI system model in calculating the magnetization amplitude and equivalent phase lag under univariate change condition: (**a**) *k* ∈ [1000 kA/m, 4000 kA/m], *a* = 12 kA/m, *c* = 0.2, *α* = −0.001; (**b**) *a* ∈ [8000 kA/m, 15,500 kA/m], *k* = 2 kA/m, *c* = 0.2, *α* = −0.001; (**c**) *α* ∈ [−0.01, 0.005], *k* = 2 kA/m, *a* = 12 kA/m, *c* = 0.2; (**d**) *c* ∈ [0.1, 0.3], *k* = 2 kA/m, *a* = 12 kA/m, *α* = −0.001.

Figure 10 shows the relative errors under fixed parameters. We extracted the maximum relative errors under more parameters (univariate change), as shown in Figure 11. According to the error analysis results, the prediction effect of the magnetization amplitude is good, as the calculation errors of the amplitudes are lower than 5%. Moreover, the precision of computing the phase lag is acceptable within the given parameter range. With an acceptable upper error limit of about 5%, the empirical equations proposed in this paper for the first-order LTI system model are approximately suitable for the conditions of *k* ∈ [1000 kA/m, 4000 kA/m], *a* ∈ [8000 kA/m, 15,500 kA/m], *α* ∈ [−0.01, 0.005], and *c* ∈ [0.1, 0.3].

**Figure 12:**
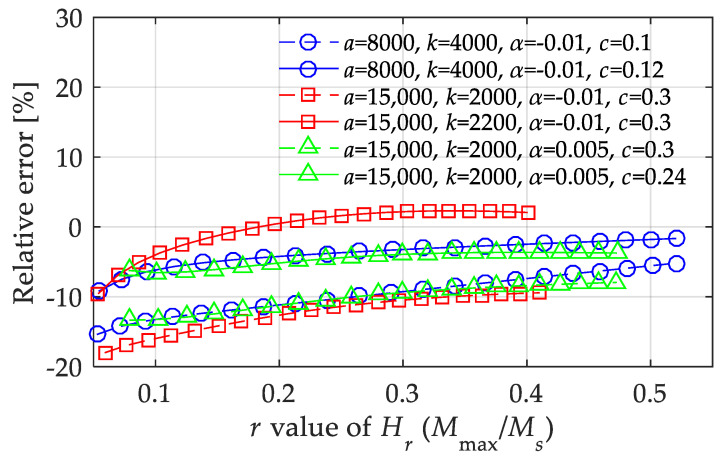
Relative errors of the 1st LTI system model in calculating the magnetization amplitude under boundary conditions with highest errors.

The relative errors in Figure 11 were obtained by changing a single parameter, and the other parameters were fixed. To implement an effective supplement for the multivariate function expression, Figure 12 shows the maximum relative errors under three boundary conditions when computing the magnetization amplitude. From the calculation results, besides the effective parameter range given above, it was determined that the value of *c* should not be too low, preferably not lower than 0.12, when the value of *k*/*a* is quite low. Moreover, the value of *c* should not be too high, preferably not higher than 0.24, when the value of *k*/*a* is quite high. Meanwhile, the value of *α* had a constraint effect on the relative error, indicating that a higher value of α is conducive to roughly averaging the errors to different values of *H*_max_.

## 6. Nonlinear Simple Function Sub-Model under High Magnetic Field Intensity

### 6.1. Optimal Parameter for Amplitude

The nonlinear simple function sub-model, similar to anhysteretic magnetization *M_an_*, is suitable when the hysteresis loop can be neglected. In the expression of $M(t)=\frac{M_{s}H(t)}{\sqrt{H{\left(t\right)}^{2}+K_{an}^{2}}}$, *K_an_* is the only parameter independent of *t* or *H* while being related to the material properties. For the exact amplitude, *K_an_* under certain materials can be easily determined from $\frac{M_{s}H_{\max}}{\sqrt{H_{\max}^{2}+K_{an}^{2}}}=M_{\max}$, with the predetermined values of *H*_max_ and *M*_max_ indicating that

(10) $${\hat{K}}_{an}=\sqrt{{\left(\frac{M_{S}H_{\max}}{M_{\max}}\right)}^{2}-H_{\max}^{2}}$$

### 6.2. Empirical Equations for the Parameters

#### 6.2.1. Function Format

Here, an empirical equation for *K_an_* suitable for various materials is provided. To determine *K_an_*, the derivatives of the nonlinear simple function and *M_an_* with respect to *H* can be used to make a comparison. The derivative function of the nonlinear simple function is $\frac{dM}{dH}=\frac{M_{s}K_{an}^{2}}{{\left[H{\left(t\right)}^{2}+K_{an}^{2}\right]}^{3/2}}$. Considering the limit value of $\underset{H\to 0}{\lim}\frac{dM_{\mathrm{an}}}{dH}=\frac{1}{3a/M_{s}-\alpha }$ in the J–A model, a rough estimate can be set as ${\left.\frac{dM}{dH}\right|}_{H=0}=\frac{M_{s}}{K_{an}}\approx \frac{1}{3a/M_{s}-\alpha }$.

By introducing a new parameter *β* to reduce the deviations of the amplitudes of *M* and *M_an_*, one obtains an empirical expression of *K_an_*:

(11) $${\hat{K}}_{an}=\beta (3a-\alpha M_{s})$$

#### 6.2.2. Parameter Determination

**Figure 13:**
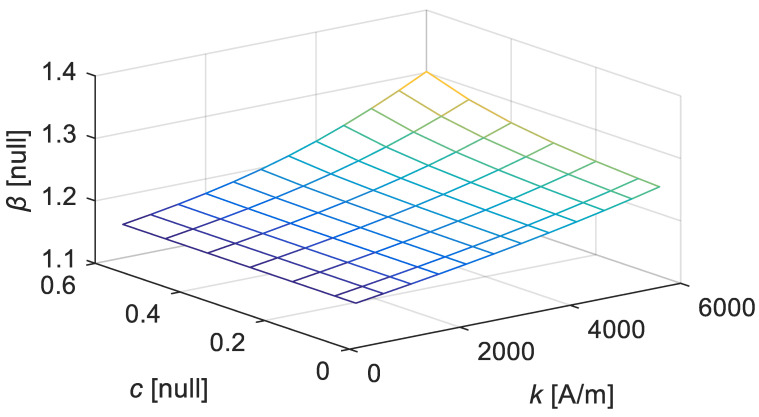
Estimates of *β* under different values of *k* and *c* (*a* = 12 kA/m, *α* = −0.001, *M_S_* = 80 kA/m).

By setting Equation (11) to be equal to Equation (10), one obtains the optimal estimate of *β* as $\hat{\beta }=\frac{H_{\max}}{M_{\max}}\frac{\sqrt{M_{s}^{2}-M_{\max}^{2}}}{3a-\alpha M_{s}}$. *K_an_* is a parameter dependent on the material property, which is represented as the five parameters of *k*, *a*, *c*, *α*, and *M_S_* in the Jiles–Atherton model. As *a*, *α*, and *M_S_* are considered in the sub-part of *K_an_*, as shown in Equation (10), it is reasonable to construct *β* using the function mainly dependent on *k* and *c*. The estimated values of *β* under different parameters are extracted, and they are plotted in Figure 13.

By constructing the multivariate function expression using a basic polynomial fitting of binary function, or by combining the univariate functions as described in Section 5.2.2 and then taking a simple function of *α* to execute a minor correction, one finally obtains the *β* expression:

(12) $$\hat{\beta }=(1+10\alpha )(2.235\times 10^{-9}k^{2}-0.02579c^{2}+1.59\times 10^{-5}ck+1.158)$$

By substituting Equation (12) into Equation (11), one obtains

(13) $${\hat{K}}_{an}=(3a-\alpha M_{s})(1+10\alpha )(2.235\times 10^{-9}k^{2}-0.0258c^{2}+1.59\times 10^{-5}ck+1.158)$$

*K_an_* is a time-independent and *H*_max_-independent parameter. By substituting Equation (13) into $M(t)=M_{s}H(t)/\sqrt{H{\left(t\right)}^{2}+K_{an}^{2}}$, a simple nonlinear expression describing the *M*–*H* relationship can be achieved and is suitable for the condition of *H*_max_ being high. Compared to the first-order LTI system model applied to the low-*H*_max_ condition, the simple nonlinear model employs the parameter *K_an_*, independent of the magnetic field intensity, and then predicting the value of *H*_max_ is no longer required. Furthermore, the influence of the frequency is not naturally considered in the nonlinear model, indicating that some frequency factors should be added to *K_an_* (or other terms) under quite a high frequency or that other conditions where the frequency has an influence should be considered.

#### 6.2.3. Verification under Fixed Values

**Figure 14:**
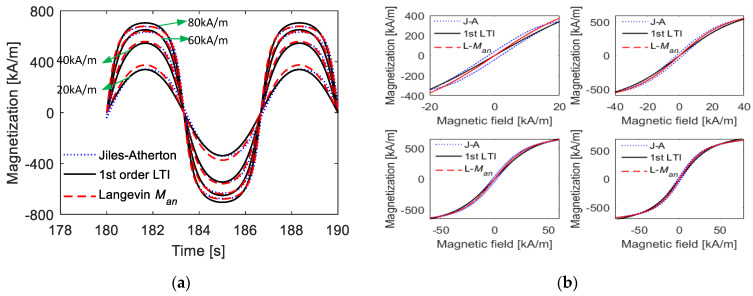
Comparisons of calculated magnetization curves from Jiles–Atherton model and the nonlinear simple model under fixed parameters (*k* = 2 kA/m, *a* = 12 kA/m, *c* = 0.2, *α* = −0.001 and *M_S_* = 80 kA/m) during various magnetic field intensities with high amplitudes: (**a**) time-domain curves; (**b**) *M*–*H* curves.

Figure 14 shows the prediction effect of the proposed nonlinear model, including time-domain curves and *M*–*H* curves under various magnetic field intensities. At the same time, the calculation effect of using *M_an_* to approximate *M* is also given. The maximum values of the magnetic field intensity are *H*_0.427_ = 20 kA/m, *H*_0.681_ = 40 kA/m, *H*_0.790_ = 60 kA/m, and *H*_0.845_ = 80 kA/m.

It can be observed from the calculation results that the model can effectively predict the amplitude and shape of magnetization when the maximum value of the magnetic field intensity belongs to *H*_0.5_~*H*_0.9_, as the maximum relative errors were only 0.98%, 0.19%, 2.99%, and 4.38%. In contrast, the maximum relative errors were 9.47%, 2.58%, 0.99%, and 0.50% when *M_an_* was used for the calculation. Moreover, with the increase in *H*_max_, the influence of the hysteresis width becomes smaller, meaning that the computation effect of *M_an_* will be better. Overall, the nonlinear function in the form of $M(t)=M_{s}H(t)/\sqrt{H{\left(t\right)}^{2}+K_{an}^{2}}$ can accurately describe magnetization under a large range with quite a simple format.

#### 6.2.4. Parameter Applicability

**Figure 15:**
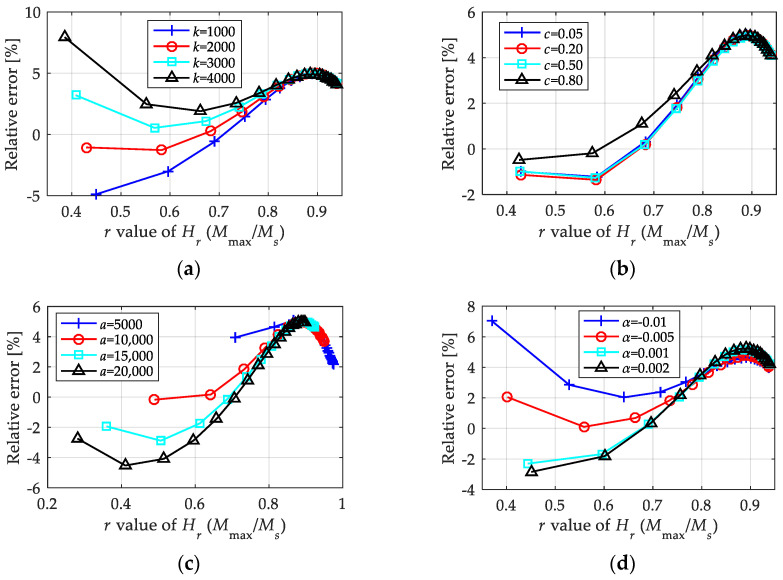
Relative errors of the nonlinear model in calculating the magnetization amplitude and equivalent phase lag under univariate change condition: (**a**) *k* ∈ [1000 kA/m, 4000 kA/m], *a* = 12 kA/m, *c* = 0.2, *α* = −0.001; (**b**) *a* ∈ [5000 kA/m, 20,000 kA/m], *k* = 2 kA/m, *c* = 0.2, *α* = −0.001; (**c**) *α* ∈ [−0.01, 0.002], *k* = 2 kA/m, *a* = 12 kA/m, *c* = 0.2; (**d**) *c* ∈ [0.05, 0.8], *k* = 2 kA/m, *a* = 12 kA/m, *α* = −0.001.

Figure 15 provides the extracted maximum relative errors under more parameters (univariate change). It can be observed from the calculation results that the proposed empirical equation exhibited good parameter applicability, as the relative errors under various parameters were low. Taking 5% as the acceptable upper limit of the relative error, the simple nonlinear model is roughly applicable to the parameter range of *k* ∈ [1000, 4000], *a* ∈ [5000, 20,000], *α* ∈ [−0.01, 0.002], and *c* ∈ (0, 1).

**Figure 16:**
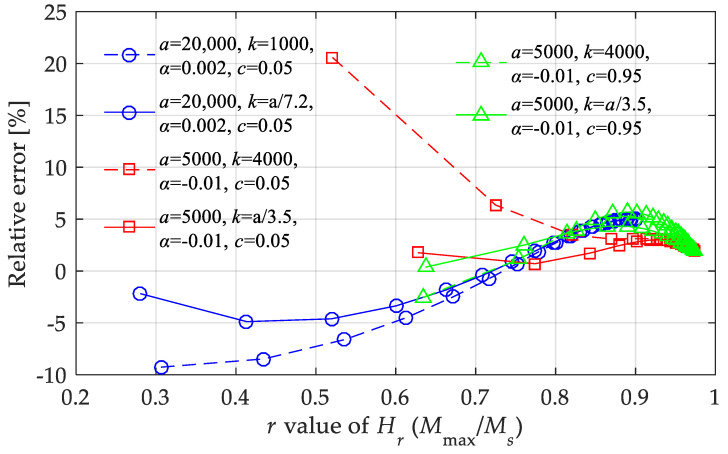
Relative errors of the nonlinear model under boundary conditions with highest errors.

Figure 16 shows the maximum relative errors under three boundary conditions to supply the error analysis. From the calculation results, besides the effective parameter range given above, the proposed nonlinear model should meet *k* ∈ [*a*/7.2, *a*/3.5] when all the parameters take extreme values (such cases are rare) and when the magnetic field intensity is not too large.

## 7. A Simple Summary

To describe *M*, Equation (6) gives the method of using the first-order LTI system under a low value of *H*_max_ and a simple nonlinear function under a high value of *H*_max_.

(14) $$\left\{\begin{array}{ll} L\frac{dM(t)}{dt}+RM(t)=M_{s}H(t), & if\;H_{\max}\leq Hr\\ M(t)=\frac{M_{s}\cdot H(t)}{\sqrt{H{\left(t\right)}^{2}+K_{an}^{2}}}, & if\;H_{\max}>Hr \end{array}\right.$$

where *L* and *R* are dependent on the material property parameters (except for *M_S_*) and *H*_max_, and *L* is dependent on an increase in *H* at the same time. When *H*_max_ ≈ *H_r_*, both sub-models are acceptable.

All the parameters can be easily determined by using the fitting method for certain materials. Another easily used determination method is given by the following empirical expressions:

(15) r=0.5L^=ALHmaxBL/ωL, R^=Hmax+CRHmaxER+DR A^L=−16k2+6.74×104k+8364a+2.55×108e−7.2c−1.17×1011α−22.68×109α−1.8×108 B^L=1.43×10−8k2+1850/a+0.21e2.7c+312α2+2α−1.5 C^R=4.86k2+0.2a2−4500a+8.65×107c3−9.56×107c2+2.13×107c−1.8×108α+9.23×106 D^R=−0.0062k2+34.5k+2.3×10−4a2−4.33a−3.23×107c10.6+5×106α+21.3×105α−2.23×104 E^R=1.28×10−7k2−8.34×10−4k−5.1×10−9a2+1.26×10−4a+2.6c3.4−0.17K^an=β(3a−αMs) β^=(1+10α)(2.235×10−9k2−0.02579c2+1.59×10−5ck+1.158)

where *ω_L_* is employed to remove the influence of the waveform and the frequency of the magnetic field intensity. Other parameters are based on the J–A model.

It should be repeated that the empirical equations shown in Equation (15) are not necessary for parameter determination. For specific materials, these parameters are fixed as α, k, etc., in the Jiles–Atherton model. Moreover, all the parameters can be determined using any fitting method or computation after extracting some important characteristic values, as described in Section 5.1 and Section 6.1. In fact, the parameters determined by the fitting method or computation after extraction can achieve better effects than the empirical equations shown in Equation (15), as they do not need to be responsible for other situations.

## 8. On/Off Type Device—A Special Example

For on/off type devices, the response is mainly determined by the amplitude and response time, while the change process from minimum to maximum has little influence, as its duration is quite short. Furthermore, the time lag (or phase lag) of magnetization compared to the magnetic field occupies a small proportion of the whole time lag, which is mainly caused by the electrical inductance, mechanical clearance, and damper. Under this condition, the first-order LTI system or the nonlinear function model is capable of describing magnetization more accurately. In fact, any model that can accurately describe the *M*_max_–*H*_max_ relationship and that can supply a low phase lag for *M* will be able to effectively predict the response of this type of device.

We took the giant magnetostrictive material used on a high-speed on/off device as an example to analyze the calculation differences between the above models. Table 2 shows the Jiles–Atherton model parameters of the giant magnetostrictive material, the first-order LTI system model parameters from the empirical equation, the first-order LTI system model parameters from direct fitting, the nonlinear function model parameters from the empirical equation, and the nonlinear function model parameters from direct fitting.

To guarantee a high-enough magnetic field intensity, a coil with a high ratio of inductance to resistance is often used. The relationship between the magnetic field intensity *H*(*t*), coil current *I*(*t*), and inputted voltage *U*(*t*) were given by a linear proportional equation *H*(*t*) = *CNI*(*t*)/*L* and a first-order linear ordinary differential equation *L_elec_*d*I*(*t*)/d*t* + *R_elec_I*(*t*) = *U*(*t*), where *C*, *N*, and *L* are proportional parameters of reluctance, the number of the winding turns, and the material length, respectively, and *L_elec_* and *R_elec_* are the inductance and resistance of the circuit with values of 50 mH and 5 Ω, respectively.

**Figure 17:**
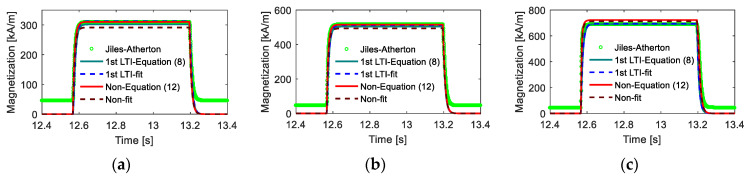
Time-domain responses calculated from different models (**a**) low magnetic field intensity, (**b**) medium magnetic field intensity, (**c**) high magnetic field intensity.

The coil was stimulated by a DC square wave with a high-voltage duration of π/5 s, and the response calculation results under different models are shown in Figure 17. From the calculated results, it was observed that the computation deviations in the change process were covered up, as the rise and fall times were quite short compared with the duration of the high magnetic field, similar to the above analysis. Moreover, the results verified the effectiveness of the proposed models, as the prediction effects of both models on the variation trend and amplitude of magnetization were acceptable.

**Figure 18:**
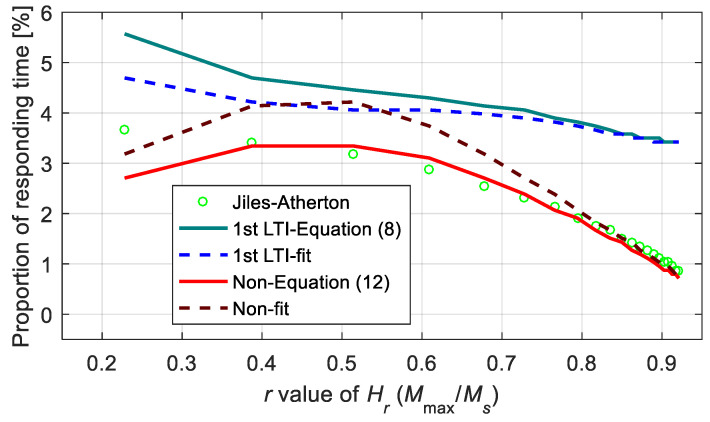
Proportions of the response time calculated from different models.

To further analyze the response time, Figure 18 shows the proportion of the magnetization response time to the high-voltage duration. The rise time was defined by the time required for magnetization to increase from 0 (or minimum) to 90% of the maximum value. From the calculations, it was observed that the response times of all the models accounted for very low proportions, less than 6%, which had small effects on the performance of the on/off type device. In addition, the response time calculated using the first-order LTI system sub-model was always slightly higher than the one calculated using the Jiles–Atherton model for the introduction of new differential elements. In contrast, the nonlinear function model did not introduce a new delay element, indicating that it was better in describing the response time.

**Figure 19:**
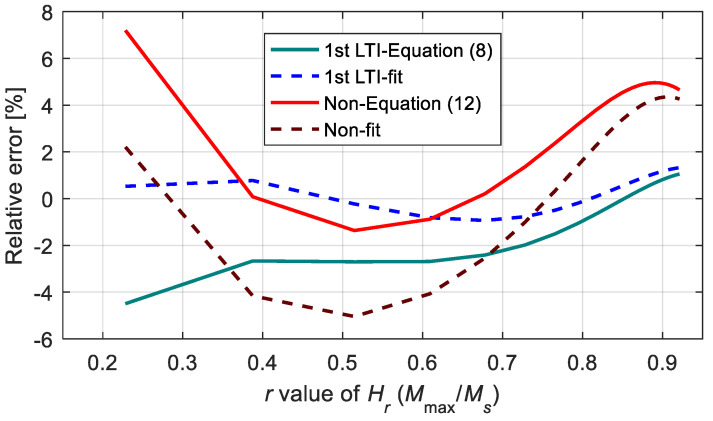
Relative errors on computing the magnetization amplitude.

Figure 19 shows the relative errors of the proposed models in computing the amplitude. According to the calculation results, as long as *H*_max_ was not less than *H*_0.25_ (this condition can always be satisfied in engineering applications), the relative errors of the two models were not more than 5%, which shows a good calculation effect of the two models. Furthermore, the first-order LTI system sub-model with direct fitting parameters had the highest calculation accuracy, and the relative errors of this method were less than 1.5%.

When describing the magnetization stimulated by the magnetic field in a DC square waveform, both the first-order LTI system sub-model and the nonlinear function model “fail” to describe the residual magnetization, which dramatically reflects the advantages of the two models used in engineering modeling. Residual magnetization is not critical, as its effect is always removed (except for materials working based on their residuals); strictly speaking, it should be removed. Residual magnetization with a low amplitude fails to overcome the preload or interstice of the devices, and it cannot produce any mechanical response, which is the same as the effect of zero magnetization. Moreover, when the residual magnetization is large enough to trigger an effective response, negative magnetic field compensation is generally required to eliminate the influence of the residual magnetization. That is, the final response of the device should be zero with any residual magnetization. To model this practical condition, the Jiles–Atherton model or other hysteresis models with a residual must be used, at least with the cooperation of a dead-zone function. On the contrary, the first-order LTI system sub-model or the nonlinear function model shows their conciseness and efficiency, as both can describe this condition without the introduction of any new sub-models.

## 9. Conclusions

The first-order LTI system model and a nonlinear function model were proposed to describe the hysteresis characteristics of a ferromagnetic material with a simple format. Moreover, the parameter determination methods and results were given based on the Jiles–Atherton model parameters.

(1) Neglecting the nonlinearity of anhysteretic magnetization, the first-order LTI system model was suitable for conditions with a magnetic field amplitude not higher than *H*_0.5_. Moreover, empirical expressions for parameters applicable to various materials were given based on univariate fitting and the linear combination of univariate functions. The proposed equations required the foreknowledge of the maximum and the increase in the magnetic field intensity, and they were approximately suitable for the parameter ranges *k* ∈ [1000, 4000], *a* ∈ [8000, 15,500], *α* ∈ [−0.01, 0.005], and *c* ∈ (0.1, 0.3). An error analysis showed good performance of the first-order LTI system model, as the calculation errors of the amplitudes were lower than 5%, and the precision of computing the phase lag was acceptable.
(2) Neglecting the phase lag, the nonlinear function model was suitable under a magnetic field amplitude not lower than *H*_0.5_. The nonlinear function model had only one parameter and did not require any foreknowledge. Empirical equations for the parameters were given and suitable for the parameter ranges *k* ∈ [1000, 4000], *a* ∈ [5000, 20,000], *α* ∈ [−0.01, 0.002], and *c* ∈ (0, 1). The error analysis showed good performance of the nonlinear function model, as the calculation errors were lower than 5%.
(3) Both the first-order LTI system model and the nonlinear function model can effectively predict the magnetization of the material employed in an on/off type device. Taking giant magnetostrictive material as an example, the two models with the parameters from the direct fitting or empirical equations showed acceptable calculation effects, as the relative errors when computing the amplitude were not higher than 5%, and the proportions of the responding time under all conditions were less than 6%. Furthermore, the first-order LTI system model with direct fitting parameters performed the best when computing the amplitude, with a relative error less than 1.5%, while the nonlinear function model was better in describing the response time when not introducing a time lag.

## Figures and Tables

**Table 1:** Calculated areas covered by the hysteresis curves from J–A model and 1st-order LTI system model.

Conditions	Area of Hysteretic Loop [kA^2^/m^2^]		Relative Error [%]
	J–A Model	1st-Order LTI Model	
*H*_max_ = 5 kA/m	350.6	337.9	3.63
*H*_max_ = 10 kA/m	1160.1	1122.9	3.21
*H*_max_ = 15 kA/m	1924.5	1898.1	1.37
*H*_max_ = 20 kA/m	2581.6	2478.5	3.99

**Table 2:** Parameters of the giant magnetostrictive material from various models or accessing ways.

Jiles–Atherton Model		1st-Order LTI Model with Empirical Parameters		1st-Order LTI Model with Fitted Parameters		Nonlinear Model with Empirical Parameters	
Parameter	Value	Parameter	Value	Parameter	Value	Parameter	Value
*α* [null]	−0.001	*A_L_* [A·s/m]	6.355 × 10^7^	*A_L_* [A·s/m]	1.933 × 10^7^	*K_an_* [A/m]	4.278 × 10^4^
*k* [A/m]	2200	*B_L_* [null]	−0.937	*B_L_* [null]	−0.807		
*c* [null]	0.18	*C_R_* [A/m]	8.973 × 10^6^	*C_R_* [A/m]	6.545 × 10^6^	**Nonlinear Model with Fitted Parameters**	
*a* [A/m]	12,000	*D_R_* [A/m]	4627	*D_R_* [A/m]	4071		
*M_s_* [A/m]	8 × 10^5^	*E_R_* [null]	−0.6	*E_R_* [null]	−0.572	**Parameter**	**Value**
		*ω_L_*	40	*ω_L_*	40	*K_an_* [A/m]	4.5 × 10^4^

**Figure A1:**
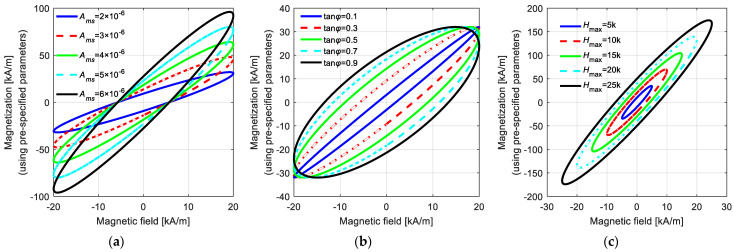
Various hysteresis loops described by the 1st-order LTI system model: (**a**) *M*–*H* curves with different values of *A_ms_*; (**b**) *M*–*H* curves with different values of *φ*; (**c**) *M*–*H* curves with different values of *H*_max_.

**Figure A2:**
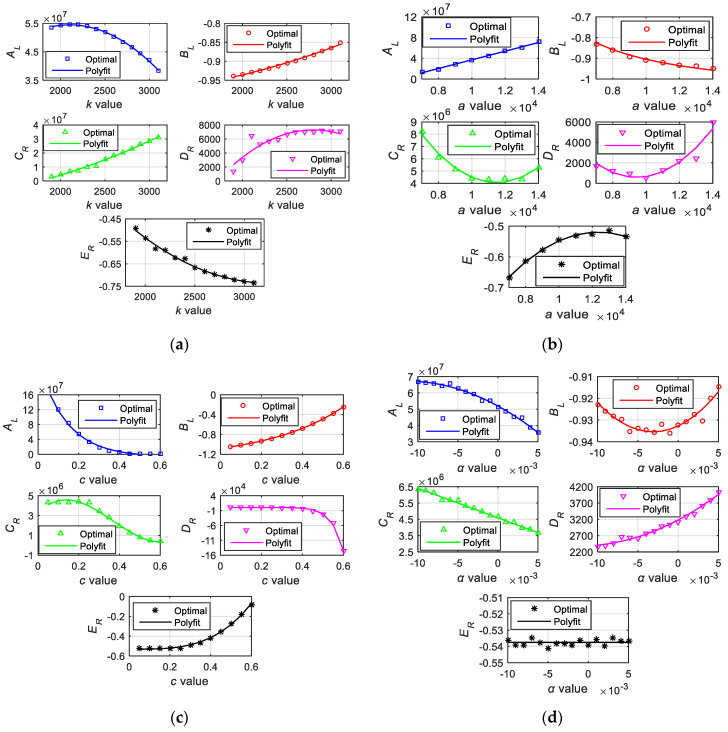
Optimal and fitted values of the parameters under univariate analysis. (**a**) take *k* as independent variable; (**b**) take *a* as independent variable; (**c**) take *c* as independent variable; (**d**) take *α* as independent variable.

## Data Availability

The data presented in this study are available in this article.

## Appendix A

Imposing *A_ms_* = $\frac{1}{\sqrt{{\left(L\omega \right)}^{2}+R^{2}}}$ to represent the ratio of magnetization amplitude to *M_S_*·*H*_max_, Figure A1a shows various *M*–*H* curves with different values of *A_ms_* under the condition that tan *φ* = 0.3(*φ* = 0.29 rad). Figure A1b shows *M*–*H* curves with different values of φ under the condition that *A_ms_* = 5 × 10^−6^, and Figure A1c shows various *M*–*H* curves with different values of *H*_max_ or *M_S_*. The calculation results show that the first-order LTI system model can effectively predict the hysteresis loop with various amplitudes, widths, and slopes (the slope of the line connecting the farthest points and the center of the hysteresis loop). However, the first-order LTI system fails to predict the hysteresis loop with a changing slope, as the mean points of the upper and lower branches of the hysteresis loop are always summarized in a straight line.

## Appendix B

Take *k* = 2 kA/m, *a* = 12 kA/m, *c* = 0.2, and *α* = −0.001 as the initial values, and change each parameter to obtain the univariate function relationship between the optimal values of *A_L_*, *B_L_*, *C_L_*, *D_R_*, *E_R_*, and the specified variable, as shown in the figures. The curves shown in Figure A2 were fitted in the simplest format possible. Taking *A_L_* as an example, from the univariate analysis, one obtains *A_L_*(*k*) = −16*k*^2^ + 6.74 × 10^4^*k* − 1.638 × 10^7^, *A_L_*(*a*) = 8364*a* − 4.657 × 10^7^, *A_L_*(*c*) = 2.55 × 10^8^e^−7.2*c*^ − 7.34 × 10^6^, and *A_L_* (*α*) = −1.17 × 10^11^*α*^2^ − 2.68 × 10^9^*α* + 5.2 × 10^7^.

Construct the multivariate function expression using linear combination, and the constant term can be obtained by substituting *A_L_*(*k* = 2000, *a* = 12,000, *c* = 0.2, *α* = −0.001) = 5.4345 × 10^7^ (or any other point), and then one obtains *A_L_*(*k*, *a*, *c*, *α*) = (−16*k*^2^ + 6.74 × 10^4^*k*) + (8364*a*) + (2.55 × 10^8^e^−7.2*c*^) + (−1.17 × 10^11^*α*^2^ − 2.68 × 10^9^*α*) − 1.8 × 10^8^.
